# Conservation of Wild Food Plants and Their Potential for Combatting Food Insecurity in Kenya as Exemplified by the Drylands of Kitui County

**DOI:** 10.3390/plants9081017

**Published:** 2020-08-12

**Authors:** Fredrick Munyao Mutie, Peninah Cheptoo Rono, Vivian Kathambi, Guang-Wan Hu, Qing-Feng Wang

**Affiliations:** 1 CAS Key Laboratory of Plant Germplasm Enhancement and Speciality Agriculture, Wuhan Botanical Gen, Chinese Academy of Sciences, Wuhan 430074, China; munyao@wbgcas.cn (F.M.M.); peninah@wbgcas.cn (P.C.R.); qfwang@wbgcas.cn (Q.-F.W.); 2 Sino-Africa Joint Research Center, Chinese Academy of Sciences, Wuhan 430074, China; 3 University of Chinese Academy of Sciences, Beijing 100049, China; 4 National Museums of Kenya, East African Herbarium, P.O. Box 40658, Nairobi 00100, Kenya; vivian@wbgcas.cn

**Keywords:** climate change, conservation, crop wild relatives (CWR), drylands, Kitui county, wild food plants

## Abstract

Wild food plants are important resources for people living in dry areas of Kenya. A botanical inventory of vascular plants of Kitui county was compiled from specimens collected during field investigations in Kitui county, at the East African (EA) herbarium and from literature reporting on plants of Kitui county. To obtain an inventory of wild edible plants found in Kitui county, literature reporting on wild edible plants of Kenya were searched and combined with the use reports obtained from field surveys in Kitui county. A total of 199 wild plants found in Kitui county have the potential of being utilized as foods in different ways. Plant species growing either as trees or shrubs (83 species) and herbs (36 species) are the dominant life forms while the best represented plant families are Leguminosae (25 species) and Malvaceae (17 species). Fruits (124 reports) and leaves (56 reports) are the common plant parts collected for food. Fruits (120 species) and vegetables (44 species) are the common wild food types in Kitui county. Further studies on species distribution are necessary to address conservation concerns that may threaten such plants.

## 1. Introduction

Arid and semi-arid areas cover about 80% of the Kenyan land mass and are characterized by a hot and dry climate and soils of poor agricultural potential [1]. The lives of people living in dry areas of Kenya are thus constrained by frequent droughts, which in turn trigger further challenges such as poor grazing resources and poor water quality. This in turn results in poverty and human to human conflicts over scarce resources. These challenges coupled with poor veterinary services make pastoralism an increasingly difficult source of livelihood for dryland communities [2]. Climate manifestation in East Africa has also proved to be difficult where warming is likely to lead to dryness in some areas and a higher precipitation in others [3], meaning that local populations are forced to cope with climatic uncertainties [4,5]. Livelihood diversification is the main way to cope with drought [6]. There is evidence of livelihood diversification in dry areas of Kenya to cope with changing climatic conditions such as the extraction of gums and resins by some communities as a source of income [7,8] while wild plants are also reported as important sources of traditional foods [9,10,11].

Many foodstuffs consumed in tropical Africa are derived from wild plants [9]. Those plants are utilized in different ways such as fruits, vegetables, cereals, roots, and tubers [11]. Plant derived foods such as fruits and nuts provide many nutritional benefits to the body [12]. Fruits can improve the nutrition of poor people who may suffer from deficiencies in vitamins, minerals, and other macronutrients. Many fruits are also important sources of vitamins A and C which may be lacking in the diet. For example, vitamin C which is found in significant quantities in many fruits is essential for protecting body cells and improves the adsorption of nonheme iron from plant-based foods. As a result of low intake of vitamin A, an estimated 50 million children in Africa are at risk of its deficiency, making it the third greatest health problem in the continent, preceded by malaria and HIV/AIDS [13]. Traditionally, children used to eat wild foods such as fruits and nuts during herding, which served them with nutritional benefits [12]. Although foods from wild plants may not at the present time form a major part of the diet of the local communities in Kenya as exemplified by the life of Dorobo people [14], traditional foods are still culturally accepted and are an integral part of the diet of local inhabitants [11]. For example, among the Dorobo people, plant species such as *Grewia tephrodermis*, *Vangueria madagascariensis*, *Vigna frutescens*, and *Vatovaea pseudolablab* are reported to have been served as staple foods for many years [14]. Furthermore, some traditional local vegetable species such as leaf amaranth are sold in the local markets in Kenya [15]. The local people have the knowledge on preparation and production of traditional foods, which require minimal additional inputs which are affordable to many, including the poor people [15]. Some wild foods also have medicinal properties to the human body and can be processed through various methods such as boiling, fermentation, and sun drying by the local people [11]. In some societies, some traditional foods from wild plants might be considered to be of no or low commercial value hence their collection is mostly meant for local consumption [11]. However, many wild indigenous fruits are sold locally in Kenyan markets such as fruits of *Adansonia digitata* which is also processed by coloring the seed pulp to make a snack. Its products are also in global demand for novel foods, pharmaceuticals, and cosmetics where the European Union, United States, Japan, and South Africa are reportedly potential markets [13].

The exploitation of native flora can be a buffer against periodical famines which are becoming prevalent in tropical areas [9]. About 60% of the Kenyan population face starvation due to lack of physical and economic access to adequate calories [16]. Kenya is endowed with diverse plant species which are estimated to comprise about 6293 indigenous vascular plants [17]. These include an estimated 800 food plants [10] some of which are underutilized food plants [16] such as *Amaranthus* spp. (leaf amaranth), *Solanum americanum* (African nightshade), *Cleome gynandra* (spider plant), *Cucumis dipsaceus* (Hedgehog cucumber), *Commelina forskaolei* (Rat’s ear), and *Cucurbita* spp. (pumpkin leaves) which can all be utilized as green leafy vegetables [15]. Such plant species were relied upon in the past as sources of vitamins, minerals, and proteins by rural societies [16]. Despite their importance, ethnobotanical knowledge of traditional wild foods is declining in Kenya [10,11]. Women, children, and herders play important roles during collection of wild edible plants [14,18] and can therefore be considered as important custodians of such knowledge in Kenya. In northern Kenya for example, collection of gums is mostly done by married women in an effort to provide an additional income for their households [8], perhaps adopting the roles of single parenthood especially the widowed. In addition, some wild food plants considered to be of minor significance are gathered by little children and are at times used as diet supplements and emergency foods [9,10]. During collection of some wild edible plants in Kenya such as *Ficus* fruits (figs), *Vangueria* fruits, *Craibia laurentii* nuts, and *Maerua kirkii* nuts, children accompany their mothers to help in gathering while collection of some species such as tubers of *Cyphia glandulosa* is reportedly done by children as they go on with their duties [14]. Collection of wild edible plants is mostly done by poor and illiterate people, where such activities have perhaps been normalized as survival strategies during the dry periods of the year when there are insufficient resources available for human survival [8]. For example, among the hunter-gatherer communities in Kenya, wild foods may comprise the main diet of the day at certain times such as during famines [10].

Introduction of exotic vegetables has diverted the focus on indigenous vegetables in Kenya [16]. Recognizing the value of wild food plants can be useful in conservation of germplasm for the future generations [9] as well as buffering against famine in the changing climatic conditions [18]. The need for the recognition of the nutritional value of traditional foods has resulted in campaigns for them to be incorporated into the rural and urban diets [16]. In spite of this, few studies in Kenya [9,14,19,20,21,22] have focused on documentation of wild food plants at local levels. In Kitui county, studies have mostly focused on documentation of medicinal plants [23,24,25] with little attention given to wild edible plants. Local utilization and acceptance of underutilized vegetable species has been reported in Kitui county where cowpeas are the most popular vegetable species [15]. The overall aim of this study is to highlight the potential of wild edible plants in Kitui county as resources which can be utilized in combatting food insecurity and famine by the rural dryland communities. The study is based on the assumption that the plant species documented as wild food plants elsewhere in Kenya but currently not yet used as such in Kitui county, have the potential to also be adopted and utilized as food plants in this region as well.

### 1.1. Study Area

**Figure 1:**
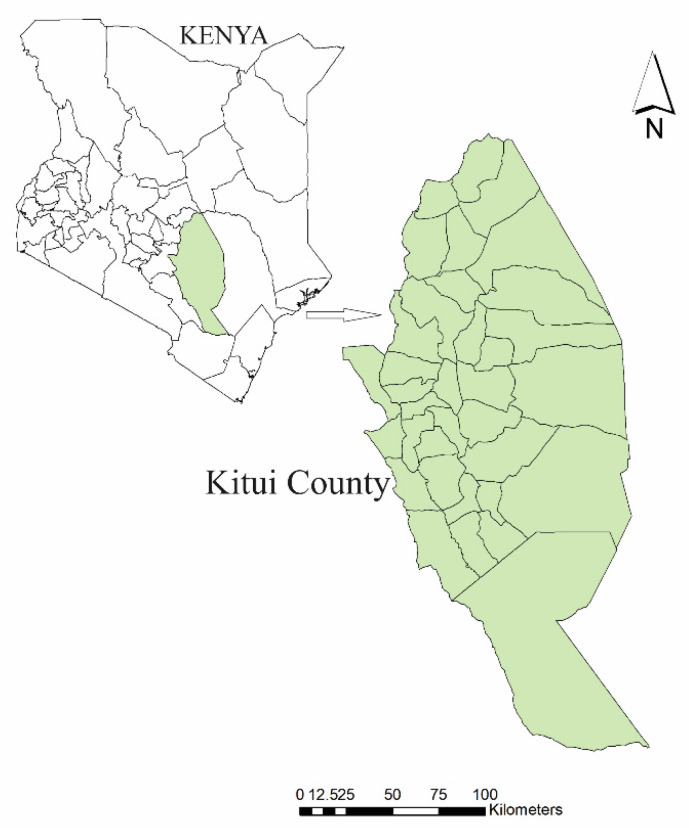
Map of Kenya showing the location of Kitui county.

Kitui county is a tract of land located at 0°10′ S and 39°0′ E, between Athi and Tana rivers occupying an area of 30,496.4 km^2^ [26,27] (Figure 1). The area is mainly inhabited by Kamba people while Tharaka people are found in the North of Tana River [26]. Kitui Kamba also interact with Oromo and Somali ethnic groups during droughts when the latter two move seeking pastures [2]. The area experiences infrequent rain and lacks permanent waters except in the Athi and Tana rivers, hence water scarcity is a major problem during the dry season. The area also lacks fertile soils hence chronic droughts and famines are major adversities to the people of Kitui [2,26,28]. As a result, the inhabitants rely heavily on forest resources especially in wetter zones near hills [2]. According to the 2009 national census, the population of Kitui county was 1,012,709 with 531,427 females and 481,282 males with a population density of 44 persons per square kilometer [27].

Kitui county is largely a low plateau rising from 300 m above the sea level through various inselbergs reaching to an altitude of about 1638 m above the sea level [28]. The highest altitudes reach about 1800 m above the sea level. The climate of Kitui county varies from arid to semi-arid with a minimum mean annual temperature varying from 14 to 22 °C and a maximum mean annual temperature ranging from 26 to 34 °C. There are two rainy seasons where the long rains start from March and end in June while the short rains fall from October to December with a mean annual rainfall of 250–1050 mm [27]. Low rainfall amounts are experienced in the extremely hot lowlands while higher rainfall amounts are experienced on the hilltops [27,28,29]. As a result, the highlands are wetter and highly populated while the dry lowlands are sparsely populated [28]. Considering the amount of rainfall received in the drylands [20] and their elevational ranges above the sea level [30], Kitui county is a typical dryland region.

The vegetation of Kitui county is characterized by low, stunted, dense thorn bushes with thick undergrowth and occasional baobab trees. Much of the area lacks vegetation except on the hills [29] where scrublands and wooded bushlands are found [31] with *Drypetes*, *Combretum*, *Vepris*, and *Croton* as the dominant species [32]. In the dry areas, the dominant vegetation is composed of *Acacia* and *Commiphora* bushlands and woodlands. The vegetation on humid and cooler hills varies and mostly include *Terminalia brownii* and *Acacia polyacantha* while exotic trees such as *Grevillea robusta*, *Cupressus* spp., *Eucalyptus* spp., and *Pinus* spp. are planted on some slopes and mountains [28]. The uncultivated and intact lands are composed of dry bushes [26]. There are several hilltops containing a high diversity of plant and animal species [32]. Such highlands reportedly provide a link between coastal forests and the Kenya highland forests, resulting in the presence of unique species adapted to each individual highland [18,32]. The plant diversity in Kitui county is high and is used for traditional foods, teas, medicines among other uses by the local communities [18,28].

### 1.2. Food Plants of Kitui County

The people of Kitui practice mixed farming which involves growing a variety of crops and keeping livestock [18,28] where cattle are kept as a security against famine [33]. In 2009, 80% of the county population was reported to rely on agriculture for economic income [27]. Kitui county is one of the regions which has a high diversity of local foods in Kenya including cultivated food crops. Some vegetable species used by the local communities include African nightshade, cowpeas (*Vigna unguiculata*), *Commelina forskaolii*, leaf amaranth, spider plant, *Cucumis dipsaceus*, and pumpkin leaves [15]. The main food crops cultivated include millet, sorghum (*Sorghum bicolor*), lablab beans (*Lablab purpureus*), pigeon peas (*Cajanus cajan*), cowpeas, maize (*Zea mays*), and green grams (*Vigna* spp.) while mangoes (*Mangifera indica*) are among the cultivated fruits. In some cases, global vegetables such as tomatoes and other green leafy vegetables are also cultivated in addition to the aforementioned traditional vegetable species. Wild fruits eaten include *Adansonia digitata* (baobab), *Grewia villosa*, *Vitex doniana* (Black plum), *Lannea alata*, *Uvaria schefleri*, *Berchemia discolor*, *Azanza garckeana*, *Tamarindus indica* (Indian date)*, Vangueria madagascariensis* (Spanish tamarind), and *Cordia monoica* (Sandpaper saucer-berry) [2,15,18,28]. Goods sold in Kitui local markets during the colonial period include cultivated grain crops, sugarcane, and unspecified vegetables [34].

## 2. Results and Discussion

### 2.1. Diversity of Edible Plants

**Figure 2:**
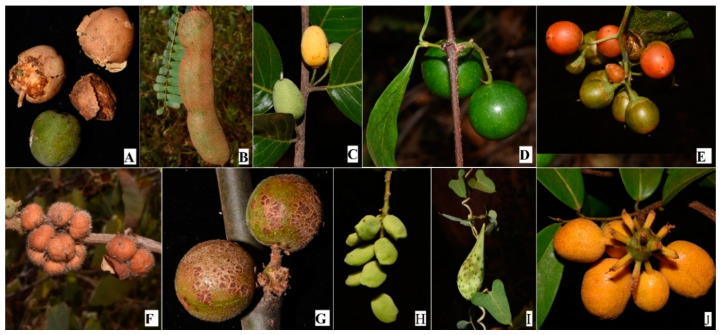
Some of the wild edible fruits encountered during field study in Kitui county: (**A**) *Balanites aegyptiaca* (L.) Delile (Zygophyllaceae); (**B**) *Tamarindus indica* L. (Leguminosae); (**C**) *Berchemia discolor* (Klotzsch) Hemsl. (Rhamnaceae); (**D**) *Vangueria madagascariensis* J.F.Gmel. (Rubiaceae); (**E**) *Cordia sinensis* Lam. (Boraginaceae); (**F**) *Grewia villosa* Willd. (Malvaceae); (**G**) *Commiphora edulis* (Klotzsch) Engl. (Burseraceae); (**H**) *Lannea schweinfurthii* Engl. (Anacardiaceae); (**I**) *Cynanchum hastifolium* K.Schum. (Apocynaceae); (**J**) *Uvaria scheffleri* Diels (Annonaceae).

A total of 199 plant species in 52 families and 114 genera currently growing in Kitui county have been documented as wild food plants in different parts of Kenya (Table 1). Some of the common wild edible fruits reported during the field work are shown (Figure 2). Leguminosae is the best represented plant family (25 species in 13 genera) followed by Malvaceae (17 species in six genera). Previous studies have reported Leguminosae to be the largest plant family in the flora of various parts of Kitui county [25,32]. It is also the largest plant family in the flora of Kenya [17]. In addition, it has been recorded to comprise most of traditional food plants utilized elsewhere in Kenya [14]. Legumes are important food plants in poor rural African communities where they provide proteins, essential amino acids, macronutrients, minerals, and vitamins. African legumes are also tolerant to drought and are therefore strategic food sources in arid areas especially under the current climatic fluctuations. Despite this, African legumes are poorly studied, and some important economic species are still obtained from the wild [35].

### 2.2. Growth Habit

**Figure 3:**
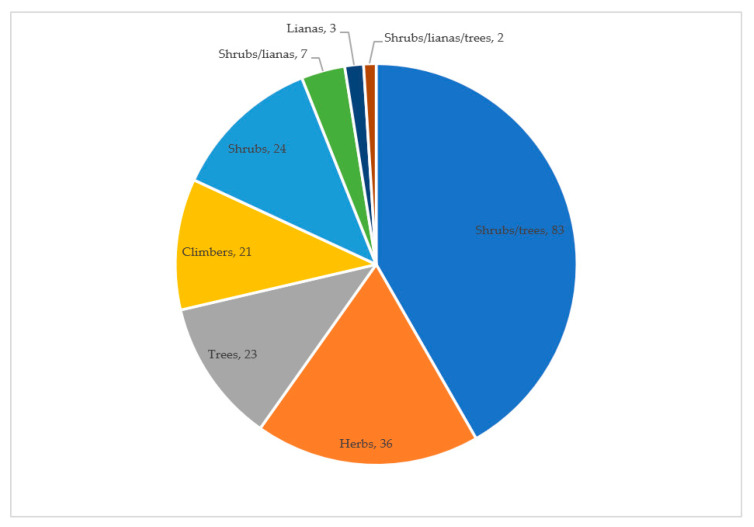
Growth habits of the wild edible plants in Kitui county.

Wild edible plant species growing as either shrubs or trees are the dominant life forms (77) recorded followed by herbs (32) and trees (21) (Figure 3). A recent ethnobotanical survey in Kitui county reported shrubs and trees to be the frequent medicinal plants reported by herbalists [25]. The vegetation of Kitui is also characterized by bushlands and woodlands composed of low, stunted thorn bushes and under-growths [28,29]. Such vegetation types are likely to be dominated by shrubs or trees.

### 2.3. Plant Parts Used

**Figure 4:**
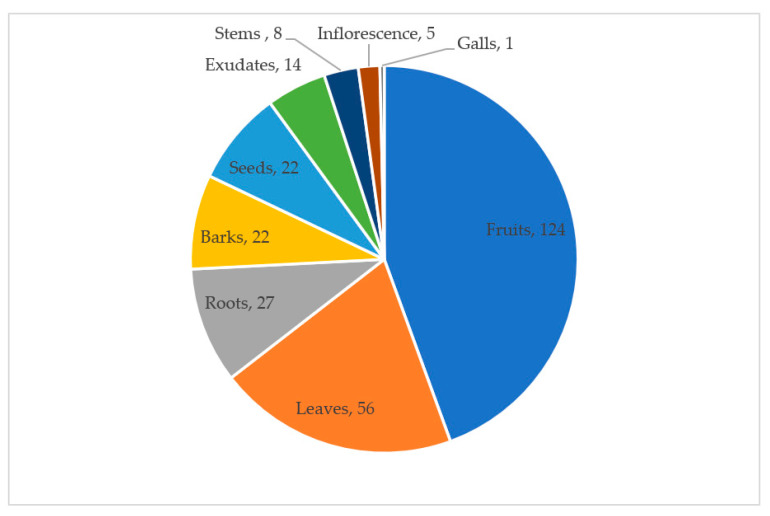
Number of species by parts of plants eaten (numbers represent reports per category).

Fruits comprise the majority of plant parts utilized as food (124 reports), followed by leaves (56 reports) while roots and barks are also frequently reported. Other plant parts such as flowers and galls are sparingly reported. A single plant may have different parts collected for food; hence such species are represented by more than one report (Figure 4).

### 2.4. Food Types Obtained from Wild Edible Plants

Foods obtained from wild edible plants reported in Kitui county fall into different categories, where the best represented food types are fruits (120 species), vegetables (44 species), and beverages (28 species) (Table 2).

Fruits are reported to be eaten raw, cooked, or used in preparation of beverages such as wine and beer. They are also used as food additives such as flavoring agents in foods and soups or as fermenting agents in preparation of local brews. Fruits are among the frequently utilized wild edible plant parts in rural areas of Kenya [9,22]. Consumption of fresh fruits is beneficial to the body since they provide the body with resources such as mineral salts, vitamins A and C, carbohydrates, natural sugars, and water [12]. Some fruits are also consumed as snacks in some rural parts of Kenya [9] while in some regions, some wild fruits are considered to be of little nutritional value and therefore consumed as supplementary and emergency foods [9,28]. Utilization of wild fruits is at times constrained by some fruit plants being widely dispersed in their natural habitats making it difficult to gather enough while other plants produce small fruits which may also be unpalatable [9]. In Kitui county, some wild and cultivated fruits complement each other, where the ripening seasons alternate successively, maintaining a continued supply of fruits to the local communities. The dependence on wild fruits is reported to be higher in drier lowland areas of Kitui where cultivated fruit species are few [28]. Such areas also experience low amounts of rainfall [27,28,29], making wild fruits an important part of the local diet. In addition, fruits of *Adansonia adansonia*, *Vitex doniana*, *Azanza garckeana*, *Tamarindus indica*, and *Vangueria madagascariensis* are sold at the local markets of Kitui [18]. During the field study at Mutomo subcounty, fruits of *Berchemia discolor* were also reported to be collected and sold at Mutomo market.

Leaves are mostly utilized as green vegetables and as food additives in preparation of tisanes while in some cases, sour leaves are chewed raw. Germinating seeds are also eaten raw or cooked as vegetables [36]. Leafy vegetables are major contributors to local diets of rural populations and are also abundant in local markets. It is likely that they provide similar nutritional composition as cultivated vegetables such as vitamins and minerals and are also of medicinal value to the body [9,12,16]. In Kitui county, deficiencies of vitamin A and zinc are reportedly widespread [15] hence leafy vegetables can play an important role in the diet of the local inhabitants. An advantage of picking wild vegetables is that they provide an opportunity to pick a variety of different plant taxa which in turn offers a diversity in the dietary composition compared to cultivated green vegetables. A single diet of wild vegetables may comprise of different plant taxa thus ensuring maximum nutritional benefits to the body [21]. However, some vegetable plant species bear small leaves, while others are bitter. In addition, nutritional composition and palatability of vegetables vary with season [21]. Combination of such characters mean that wild vegetables require skills and time in their preparation which might result in their avoidance [16]. Similar to cultivated vegetables that are mixed together during times of scarcity, wild vegetables are also mixed to gather enough [9]. Wild vegetables are also mixed with cultivated vegetables to improve the taste [21]. Preparation of wild vegetables may involve boiling to wash them before cooking begins, probably as a way of dealing with bitterness in some vegetable species such as *Solanum americanum* which may contain toxic alkaloids. Bitter tasting or toxic populations of wild vegetables can also be avoided during the time of picking in the wild [9,21]. Leafy vegetables can be obtained from natural habitats such as forests and in disturbed places including farmlands [21]. Cowpeas are the main vegetables in Kitui county while other vegetables are underutilized [15] or used in the absence of cowpeas [28]. For example, *Commelina africana*, a wild vegetable which grows in the farm and in the wild sprouts earlier after the rains, providing an early vegetable before maturation of cowpeas [28]. Some species of wild vegetables such as *Amaranthus graecizans*, *Solanum americanum*, and *Cleome gynandra* occur naturally including in disturbed habitats although their occurrence depends on the right season which coincides with rains [9,21]. Other leafy vegetables such as *Oxygonium sinuatum*, *Commelina africana*, and *C. benghalensis* may occur as invasive weeds in cultivations [9]. Such adaptations to the local environments make indigenous vegetables suitable candidate species for combatting food insecurity by poor people living in dry areas of Kenya [16]. Some wild vegetable plants, which are utilized in other parts of Kenya grow naturally as weeds and may be underutilized by the local communities in Kitui county. Nutritional education and cooking demonstration of underutilized wild leafy vegetables was reported to result to an increase in their utilization in Kitui county [15]. According to Ichikawa et al. [37], major food plants might be shared between different communities while minor food plants may vary from one community to another. Local people are cautious with trying cultivation of vegetables they are not familiar with [15]. To enhance diversification of the ways of obtaining foods by the local communities, it is therefore important to create awareness on the utilization of local food plants not known or less prioritized by the local communities.

Exudates comprise of gums, resins, and wines tapped from plants. Gums and resins are produced by plants throughout the season including during the dry periods of the year. Although most of such exudates are collected from plants in arid and semi-arid areas that are of poor agricultural potential, only small quantities are meant for domestic consumption and much of the material is collected for sale [8]. However, gums and resins are still locally eaten during food scarcity and also have medicinal benefits [38]. Their collection is mostly carried out during the dry season by women and children in poor communities or by opportunists interested in income generation. Harvesting of gums and resins is a viable alternative for strengthening livelihood diversification in the drylands [38] especially during the dry periods of the year when other sources of livelihood such as dependence on livestock resources are constrained by insufficient pasture [8]. Despite the important role played by gums and resins in the lives of the local communities, collectors encounter various challenges such as poor harvesting methods, contamination of the collected materials, and improper post-harvest handling techniques resulting in overall reduction of the quality of the end products and hence low prices in the market. These coupled with the poor markets where the collectors mostly sell to the local shops and further complicated by the presence of local agents and opportunist buyers makes income generation from gums and resins unsustainable. The potential of gums and resins in alleviating poverty in dry areas of Kenya are hence underutilized [8]. Development of better markets for gums and resins would be an important step towards maximizing the benefits of such products to the local communities in dry areas of Kenya including in Kitui county.

Barks are reported to be used in preparation of tisanes or as food additives such as flavorings. Raw roots are eaten as starch foods, cooked as vegetables, or used as food additives. Roots and tubers are important sources of energy since they are rich in starch. Freshly harvested roots also contain a large water content [12]. Roots and stems from some plant species are sweet and succulent hence they are chewed raw to quench thirst. In some instances, roots serve as immediate sources of food especially during grazing when the herders have little or no time to cook. Such methods of utilizing wild plants are important attributes for people to survive in dry areas [20]. Some root tubers are cooked to reduce the poisonous compounds that may be present while others are prepared through drying and pounding before consumption [9,20,36]. For example, the roots of *Thilachium africanum* are poisonous but edible when cooked [39]. Stems of some plant species such as *Albizia amara* are used as food additives, which is boiled in soup and also used as a meat tenderizer. Other plant parts reported are flowers which are eaten raw or picked with leafy parts and prepared together as vegetables, while the internal parts of galls are eaten raw. Wild edible seeds reported include pulses (seeds from legumes), cereals (seeds from grasses), pseudo-cereals (non-grass seeds that serve a similar purpose as cereals), and other seeds which are prepared through boiling, roasting, or eaten raw. Cereals and pseudo-cereals are ground into flour which is made into other dishes such as porridges. Legume seeds are important sources of proteins, iron, niacin, and vitamins hence are used as meat substitutes while other seeds are sources unsaturated fats, vitamins, and minerals such as phosphorus, calcium, and fluorine [12].

Beverages such as beer, wine, and tisanes are also prepared from wild plant parts. Tisanes include both infusions and decoctions taken as beverages, bitter teas, teas with essential oils, stimulant teas, and medicinal teas [40]. Utilization of herbal teas is dated back to the medieval medicine when they were used for therapeutic purposes [41]. Therapeutic classification of herbal teas in Kenya was also done by Ichikawa [14] who referred to them as narcotics and herbal medicines. However, Maundu et al. [36] treated some of them as foods. In this study, infusions and decoctions prepared and taken in place of caffeinated drinks and herbal additives added into caffeinated drinks are categorized as tisanes. They are prepared from the leaves and barks while in some instances, seeds are used. Tisanes are mostly prepared from plant families (Verbanaceae, Lamiaceae, Rutaceae, Burseraceae, and Anacardiaceae) comprising of aromatic, glandular, or resinous and oil producing species [39,42,43] although some species in Leguminosae, Sapindaceae, and Rhamnaceae families are also used. According to Maundu et al. [36], plants with essential oils such as *Ocimum* species are used for flavoring tea. These plants serve both as flavoring agents and as substitutes for caffeinated teas. For example, during a field survey at Mutomo subcounty in Kitui, leaves of *Zanthoxylum chalybeum* were reported by the local residents to add a good flavor to caffeinated teas while the bark of *Acacia nilotica* was said to be used as a substitute for caffeinated teas. Stem bark of *Acacia nilotica* is also reported to be a stimulant [44]. Further studies are needed to determine the role of the reported plant species in preparation of tisanes since such preparations may be categorized as flavorings, teas, coffee substitutes, and as herbal medicines. Some plant species are used in making beverages such as herbal beer and wine. These include the fruits of *Hyphaene compressa* which contain a liquid that is brewed into beer and the fruits of *Cordia sinensis* and *Balanites rotundifolia* which are used in preparation of local brews [36]. Palm exudate liquid, tapped from the vascular bundles of *Phoenix reclinata*, is also drunk as wine [45].

Food additives are also obtained from edible plants. These include spices, herbs, and seasonings which are of small nutritive value hence consumed in small amounts to stimulate appetite by enhancing flavor [12]. 

An inventory of the wild edible plants in Kitui county is provided in Table 3. Those plant species which at present are already used as wild food plants are marked with an asterisk (*) and a number sign (#).

### 2.5. Potential of Crop Wild Relatives (CWR) in Kitui County

Crop wild relatives (CWR) form an important part of gene pool for the improvement of cultivated crops [55]. The genetic relationship between many of the tropical CWR and the cultivated crops is unknown [56]. In Kenya for example, wild sorghum populations are reportedly widespread in various habitats such as in protected areas, roadsides, and farmlands. Such resources are regarded as weeds in farmers’ fields and are facing the risk of genetic contamination through pollen-mediated crop-wild introgression [57]. The negligence of CWR and land races from the notion that they will remain to be readily available in the wild is causing their degradation [58]. Some of the wild plants utilized as wild foods in Kitui county that have cultivated relatives in the area include *Amaranthus* species such as *A. dubius* [15]. The Amaranthaceae family also exhibits the highest diversity of species used as traditional vegetables in Kenya [51], hence such group of plants form an important gene pool for future improvement of cultivated members. The leaves of *Vigna membranacea* (traditional vegetable) are reported to taste similar as cultivated *V. unguiculata*, a species composed of various subspecies and several cultivars in Kenya [36]. *Vigna unguiculata* is also the second most popular grain legume in Kenya after beans, and it is estimated that 85% of the area under its cultivation in Kenya lies in arid and semi-arid areas [51]. In Tharaka for example, an arid area adjacent to Kitui, cowpeas are cultivated by about 80% of the households [59]. Pigeon pea (*Cajanus cajan*) is also an important crop in dry areas although its diversity is limited to only one species [51,59]. *Cajanus cajan* is regarded as an indigenous plant in Kenya [43], hence wild forms might form an important resource base for improvement of cultivated members especially in dry areas such as Kitui county. Other important cultivated plant species with wild forms in Kitui include *Lagenaria siceraria* and *Citrullus lanatus* [28,36,51]. *Solanum americanum* is also a vegetable species growing in the wild and cultivated in Kitui county [15]. Some of its wild forms are bitter tasting and hence avoided during vegetable collection [9,21]. Such forms might be neglected leading to their possible disappearance in the wild. Although the socio-economic importance of CWR is well known, their conservation has not been systematically addressed and their current extinction levels might result in serious social and economic problems if threats facing them are not adequately addressed [58]. In Kenya, the decline of plant genetic resources is at its peak following the effects of global warming, increased population, and desertification [51]. Conservation efforts of such critically important group of plants is therefore vital if they are to be relied upon in the future [60]. Since it is evident that drylands of Kenya harbor wild plants with a potential to combat food insecurity as exemplified by Kitui county, collection of CWR and other food plants’ germplasm and its conservation are important steps towards ensuring maximum benefits from such resources.

### 2.6. Conservation of Natural Habitats in Kitui County

Availability of wild food plants depends on the ecology of a given area and the history of its deforestation [9]. In Kenya, there is an ongoing loss of wild food species and the traditional knowledge associated with them especially in areas of high agricultural potential, where much of the original vegetation has been cleared for agriculture and infrastructure [10]. Domestication of some wild vegetables is however reportedly ongoing in some regions where vegetable plants such as *Cleome gynandra*, once introduced continue to self-reseed in subsequent years [9] hence becoming a long-term source of leafy vegetables. Other vegetables species under domestication in Kenya include *Amaranthus* spp., *Solanum americamum*, *Basella alba*, and *Sesamum angustifolium* which may also be spared in the farmland during cultivation of weeds [10]. Many of the food plants occur in natural forests while some are preserved by the local inhabitants in their farmlands. In Kitui county, fruit plants such as *Tamarindus indica* and *Balanites aegyptiaca* are preserved in farmlands for their medicinal uses [25]. Wild fruit trees are also left standing when other plants are being cleared for farmlands or charcoal. Wild food plants of Kitui county are threatened by the local communities who cut them for charcoal, thus also leading to loss of indigenous knowledge associated with them [28]. According to Mutie et al. [25], some medicinal plants in Kitui county such as *Strychnos henningsii* and *Vepris simplicifolia* which are also reported as food plants are decreasing in the wild as a result of human activities. Wild food plants are most important to the communities who reside in dry areas, which are more vulnerable to droughts [9]. Such areas are mostly inhabited by pastoral groups whose major threat to plant diversity is overstocking [10]. Diversity of wild edible plants is also reported to be richer in savanna zones compared to other forests zones [14]. It is also in the drier regions where the vegetation has been conserved to the greatest extent in some regions of Kenya [9]. The hills of Kitui are perceived by the local people to harbor important medicinal and food plant species [18,23]. In addition, high plant diversity and species endemism are reported in the hills of Kitui [32]. Such hills are vital ecosystems for adaptation towards the changing climatic conditions through provision of important ecosystems goods such as wild foods [18]. Mutomo hill plant sanctuary, one of the hills in Kitui county has been recently reported as a potentially important area for conservation of medicinal plants [25]. Conservation of important plants including wild food plants in other hills of Kitui county needs assessment and prioritization through community awareness so as to ease pressure exerted on wild plant populations by the local communities.

## 3. Materials and Methods

A botanical inventory of vascular plants of Kitui county was first compiled from data collected during three different botanical surveys in various parts of Kitui county between May 2018 and February 2019 by the Sino-Africa Joint Investigation Team (SAJIT). These include an ethnobotanical survey of medicinal plants carried out in Mutomo subcounty [25], which included citations of wild edible plants by the respondents. Further floral surveys were carried out in Endau hills, Mutitu hills, and Mui basin where the local people cited the wild plants used as food whenever they encountered them. Where possible, specimens at the EA herbarium in Kenya were checked to obtain plant species previously collected from Kitui county. Voucher specimens reported in this study have been deposited at Hubei Institute of Botany (HIB) herbarium in China and at the EA.

The data was supplemented by other data obtained from various literature such as published articles, conference proceedings, botanical survey reports, and the monographs of the Flora of Tropical East Africa reporting on plants of Kitui county (voucher materials are represented by specimen numbers seen in literature or by references citing the presence of the reported plant species in Kitui county). This yielded a plant checklist totaling to 931 vascular plant species, the most comprehensive checklist of the region to date (unpublished results). To obtain an inventory of wild edible plants of Kitui county, literature reporting on wild edible plants of Kenya was searched from various sources and combined with the use reports obtained from field surveys. Data were searched using key words ‘plants, flora, edible plants, wild fruits, fruits, livelihood diversification in drylands, vegetable plants, nuts and seeds, useful plants, edible tubers, wild teas.’ The key words were combined with ‘Kitui’ and ‘Kenya’, each at a time in order to determine the area of data collection. To exhaust the information gathered, if a plant species was found to be edible in Kenya and not yet recorded in Kitui county, another search category was initiated (‘Kitui county’ plus ‘scientific name the plant’). The indigenous plant species and their growth habits were determined based on the local monographs of Kenyan flora [39,43] and the monographs of the Flora of Tropical East Africa [42]. All plant name synonymies were resolved using The Plant List database (http://www.theplantlist.org/). The plant species were then classified into parts utilized for food and into different food categories according to Cook [61]. The data were entered and analyzed in Microsoft Excel 2016.

## 4. Conclusions

Although further circumspection is needed before the potential adoption of these plants for food, this study nevertheless presents wild plants as important sources of food for the local communities living in dry areas of Kenya such as Kitui county. Investigation of herbarium materials and further botanical surveys are still necessary to determine the undetected food plants. The local communities have incorporated some conservation measures in their farmlands. There is still a need to sensitize them further on the need of preserving natural habitats and involve them in collecting of germplasm of edible wild plants and their relatives for ex-situ conservation. Disturbed places play an important role in human nutrition through provision of green vegetables, some of which may occur as invasive weeds. Prioritizing on proper harvesting, storage, and marketing of wild foods produced seasonally in large quantities might be an important step in maximizing the nutritional benefits of dryland communities. Lastly, understanding the distribution of ethnobotanical knowledge among individuals and the role of age, gender, and the level of education are important factors in conservation of wild edible plants in dryland areas of Kenya in general.

## Figures and Tables

**Table 1:** Number of genera and species of wild edible plants in Kitui county by family.

Family	Genus	Species	Family	Genus	Species
Leguminosae	13	25	Convolvulaceae	1	2
Malvaceae	6	17	Ebenaceae	2	2
Rubiaceae	6	11	Euphorbiaceae	2	2
Anacardiaceae	3	10	Poaceae	1	2
Cucurbitaceae	7	9	Portulacaceae	1	2
Lamiaceae	4	9	Salvadoraceae	2	2
Burseraceae	2	7	Bignoniaceae	1	1
Moraceae	2	7	Campanulaceae	1	1
Amaranthaceae	3	6	Cannabaceae	1	1
Capparaceae	3	6	Cleomaceae	1	1
Rhamnaceae	3	6	Clusiaceae	1	1
Apocynaceae	5	5	Combretaceae	1	1
Rutaceae	3	5	Menispermaceae	1	1
Verbenaceae	2	5	Nymphaeaceae	1	1
Zygophyllaceae	1	5	Olacaceae	1	1
Annonaceae	2	4	Opiliaceae	1	1
Phyllanthaceae	3	4	Pedaliaceae	1	1
Boraginaceae	1	3	Polygonaceae	1	1
Commelinaceae	1	3	Salicaceae	1	1
Compositae	3	3	Santalaceae	1	1
Cyperaceae	2	3	Sapotaceae	1	1
Loganiaceae	1	3	Solanaceae	1	1
Oleaceae	2	3	Xanthorrhoeaceae	1	1
Sapindaceae	3	3	Geraniaceae	1	1
Vitaceae	2	3	Putranjivaceae	1	1
Arecaceae	2	2	Talinaceae	1	1

**Table 2:** Classification of food types and the number of species in each type.

Food Types	Number of Species	Specific Food Type	Number of Species
Fruits	120	Eaten raw	119
		Cooked	1
Vegetables	44	Green vegetables	43
		Tuber vegetables	1
Beverages	28	Tisanes	22
		Beers	4
		Wines	1
		Coffee substitutes	1
Food additives	22	Flavoring agents	17
		Sweeteners	2
		Fermenting agents	2
		Water clarifiers	2
		Milk curdlers	1
		Meat tenderizers	2
Starch foods	21	Tubers	21
Seed foods	22	Other seeds	13
		Pulses	5
		Cereals	2
		Pseudo-cereals	2
Gums and resins	13	Eaten raw	13
Others	24	Leaves chewed raw	8
		Barks chewed raw	7
		Roots chewed raw	4
		Inflorescence eaten raw	5
		Edible cotyledon/embryo	3
		Internal juice of fruit drunk	1
		Galls	1
		Stem pith chewed raw	1

**Table 3:** An inventory of wild edible plant species occurring in Kitui county. Information given under ‘presence in Kitui county’ refers to voucher specimens collected during field work by the authors (designated as SAJIT-Mutie MU), specimens at the East African (EA) herbarium or in publications citing the presence of the species in Kitui county. The plant use information refers to records of use of the plants for anywhere in Kenya, not necessarily in Kitui county, unless the name of the species is accompanied by an Asterix (*) (indicating the plant was cited during a field survey as edible) or by a number sign (#) indicating the plant use in Kitui county was obtained from literature.

Family and Plant Species	Kamba Name	Growth Habit	Presence in Kitui County	Part Used; Use in Kenya	Source of Information
Amaranthaceae					
^#^*Amaranthus dubius* Mart. ex Thell.	W’oa, telele, terere	Herb	Lind and Agnew 5642 (EA)	Leaves; eaten as a vegetable.	[15,36]
*Amaranthus graecizans* L.	W’oa, telele, terere	Herb	Gilbert 70 (EA)	Leaves; eaten as a vegetable.	[36]
*Amaranthus hybridus* L.	W’oa, telele, terere	Herb	Gilbert 98 (EA)	Leaves; eaten as a vegetable.	[21,36,42]
*Amaranthus spinosus* L.		Herb	Gilbert 97 (EA)	Leaves; eaten as a vegetable.	[36]
*Digera muricata* (L.) Mart.	Walange	Herb	Someren 2024 (EA)	Leaves, flowers; leaves eaten as a vegetable. Flower nectar is edible.	[36]
* *Aerva lanata* (L.) Juss.	_	Herb	SAJIT-Mutie MU0268 (EA)	Leaves; eaten as a vegetable.	[36]
Anacardiaceae					
^#^*Lannea alata* (Engl.) Engl.	Kitungu, ndungu, mukolya	Shrub or tree	Kuchar 14876 (EA)	Fruits, bark; ripe fruits edible. Bark used in tea.	[18,36,39]
* Lannea schweinfurthii Engl.	Muasi, kyuasi	Shrub or tree	SAJIT-Mutie MU0271 (EA)	Fruits, bark; ripe fruits edible. Bark used for making tea.	[18,39]
^#^*Lannea triphylla* (Hochst. ex A. Rich.) Engl.	Muthaalwa, kithaalwa, kithaala, nzaala	Shrub or tree	[36]	Fruits, bark, root; ripe fruits are edible. Sweet succulent roots and bark chewed raw to quench thirst. Bark used in tea.	[18,19,20,36,39]
^#^*Searsia natalensis* (Bernh. ex C.Krauss) F.A.Barkley	Kitheu, mutheu	Shrub or tree	Evans 167 (EA)	Fruits, bark, roots, leaves; ripe fruits are edible. Bark used in tea. Roots boiled in soup. Young shoots chewed raw.	[18,36,39]
* *Lannea rivae* Sacleux	Kithaalwa, muthaalwa, kithaala, kithaalua kya kiima	Shrub or tree	SAJIT-Mutie MU0305 (EA, HIB)	Fruits, bark; ripe fruits are edible. Bark is sweet and chewed raw.	[36,39]
*Lannea schimperi* (Hochst. ex A.Rich.) Engl.	kithoona, kithauna, nthoona	Shrub or tree	[36]	Fruits, bark; ripe fruits are edible. Bark used in tea.	[36]
*Searsia tenuinervis* (Engl.) Moffett	Kitheu	Shrub or tree	[36]	Fruits, leaves; ripe fruits are edible. Young shoots and leaves chewed raw.	[36,39]
*Searsia quartiniana* (A.Rich.) A.J.Mill.	Mutheu	Shrub or tree	[36]	Ripe fruits; edible.	[18,36]
*Searsia pyroides* (Burch.) Moffett	Kitheu, mutheu, mutheu munene	Shrub or tree	[36]	Ripe fruits; edible.	[36,39]
^#^*Sclerocarya birrea* (A.Rich.) Hochst.	Muua, muuw’a, mauw’a	Tree	Bogdan AB4379 (EA)	Fruits, seeds; ripe fruits are edible. Internal seed contents eaten raw.	[18,36,39,46]
Annonaceae					
^#^* *Uvaria scheffleri* Diels	Mukukuma	Shrub or liana	SAJIT-Mutie MU0290 (EA)	Ripe fruits; edible.	[18,19,36,39]
^#^*Annona senegalensis* Pers.	Makulo, mutomoko ‘wild custard apple, wild soursop’	Shrub or tree	[36]	Bark, fruits; ripe fruits are edible. Bark chewed raw.	[39,47]
^#^*Uvaria acuminata* Oliv.	_	Shrub or liana	Mwachala et al., 476 (EA)	Ripe fruits; edible.	[36,39]
*Uvaria lucida* Bojer ex Benth.	_	Shrub or liana	Mbonge 14 (EA)	Ripe fruits; edible.	[36]
Apocynaceae					
* *Saba comorensis* (Bojer ex A.DC.) Pichon	Kilia, kiongwa, kyongoa, mongoa	Liana	SAJIT-Mutie MU0278 (EA)	Ripe fruits; edible.	[22,36,39]
* *Cynanchum hastifolium* K.Schum.	_	Climber	SAJIT-Mutie MU0261 (EA)	Unripe fruits; edible.	[19]
^#^*Carissa spinarum* L.	Mukawa, mutote, ngawa, ndote, nzunu	Shrub	[48]	Flowers, fruits, roots; flowers and ripe fruits are edible. Boiled roots eaten as vegetables and used as a flavor in soup.	[18,19,20,36,39,47]
*Acokanthera schimperi* (A.DC.) Schweinf.	Kivai	Shrub or tree	[36]	Ripe fruits; edible.	[18,36,39]
* *Pentarrhinum insipidum* E.Mey.	_	Climber	SAJIT-Mutie MU0139 (EA)	Fruits, leaves; leaves eaten as a vegetable. Ripe fruits are edible.	[20,36]
Arecaceae					
*Phoenix reclinata* Jacq.	Makindu ‘wild date palm’	Tree	[36,48]	Fruits, stem; ripe fruits are edible. Wine is tapped from stem.	[36,39]
^#^*Hyphaene compressa* H.Wendl.	Mukoma, ilala	Tree	[36]	Fruits, leaves; seedling embryo is edible. Fruit pulp eaten raw. Juice from immature fruits drunk fresh or used to make beer.	[36,39,47]
Bignoniaceae					
^#^*Kigelia africana* (Lam.) Benth.	Kiatine, muatine ‘sausage tree’	Shrub or tree	[48]	Fruits; used for fermenting traditional beer.	[18,39]
^#^*Cordia monoica* Roxb.	Muthii, kithei, nthei	Shrub or tree	[48]	Ripe fruits; edible.	[18,19,36,39,47]
^#^* *Cordia sinensis* Lam.	Muthea, kithea, muthei-munini, kithia	Shrub or tree	SAJIT-Mutie MU0292 (EA)	Exudate, roots, fruits; roots eaten raw. Ripe fruits are edible. Fruit pulp used for brewing local beer. Produces an edible gum.	[18,19,36,39]
*Cordia crenata* Delile	_	Shrub or tree	Kirika et al., GBK2/10/2005 (EA)	Ripe fruits; edible.	[39]
Burseraceae					
^#^* *Commiphora baluensis* Engl.	Itula, mutula	Tree	SAJIT-Mutie MU0254 (EA)	Bark; used in making tea.	[18,19,39]
* *Commiphora edulis* (Klotzsch) Engl.	Kyoa kika	Shrub or tree	SAJIT-Mutie MU0193 (EA)	Fruits; seed aril from ripe fruit is edible.	[19]
^#^*Boswellia neglecta* S.Moore	Kinondo	Shrub or tree	Bally B1612 (EA)	Exudate, bark; resin from bark chewed. Bark used for making tea.	[18,36,39,49]
*Commiphora campestris* Engl	_	Tree	[48]	Exudate; produces an edible resin.	[49]
* *Commiphora africana* (A.Rich.) Endl.	Kitungu, mutungu, itula	Shrub or tree	Kuchar 15067 (EA)	Exudate, roots, bark; produces an edible gum. Roots of young plants chewed raw to quench thirst. Bark used for making tea.	[18,36,39]
*Commiphora rostrata* Engl.	Inywamanzi	Shrub or tree	[48]	Bark, leaves, stem; leaves chewed raw or cooked to add flavor in foods. Bark used for tea. Stem pith and bark of young plants chewed raw to quench thirst.	[18,20,36,39]
*Commiphora schimperi* (O.Bergman) Engl.	Mutungu	Shrub or tree	[36,48]	Exudate, roots, bark; produces an edible resin. Roots chewed to quench thirst. Inner red bark boiled in tea.	[19,36,39]
Campanulaceae					
^#^*Cyphia glandulifera* Hochst. ex A.Rich.	Ngomo	Herb	[36]	Roots, leaves; leaves eaten as a vegetable. Tubers eaten raw.	[14,19]
Cannabaceae					
*Trema orientalis* (L.) Blume	_	Shrub or tree	[18]	Ripe fruits; edible.	[39]
Capparaceae					
^#^*Boscia coriacea* Graells	Isivu	Shrub or tree	SAJIT-Mutie MU0122 (EA)	Fruits, seeds; fruits are edible. Seeds edible when boiled.	[18,36,39,47]
^#^*Maerua decumbens* (Brongn.) DeWolf	Kinatha, munatha	Herb or shrub	Kuchar 15244 (EA)	Roots, fruits, seeds; ripe fruits eaten raw or cooked. Seeds edible when boiled. Root bark chewed raw. Roots added to water as a sweetener.	[18,36,39,47]
^#^*Maerua denhardtiorum* Gilg	Itembokambola	Shrub	Kuchar 14991 (EA)	Ripe fruits; edible.	[18,19,39,47]
*Maerua kirkii* F. White	Ivovotwe	Shrub or tree	Kimani 86 (EA)	Nuts; boiled and eaten.	[19]
*Thilachium africanum* Lour.	Mutunguu	Shrub or tree	Greenway 9228 (EA)	Roots; cooked and eaten.	[39]
*Thilachium thomasii* Gilg	Kitungulu	Shrub or tree	Spjut and Muchai 4655 (EA)	Roots, fruits; ripe fruits are edible. Tubers eaten or cooked and the resultant liquid drunk or used for making tea. Peeled roots used as flocculants in water.	[36,39]
Cleomaceae					
^#^*Cleome gynandra* L.	Mwianzo, mukakai, sake, mwaanzo, ithea-utuku	Herb	Hucks 341 (EA)	Leaves; eaten as a vegetable.	[19,21,36,42]
Clusiaceae					
^#^*Garcinia livingstonei* T.Anderson	Mukanga, kikangakanywa, ngangakanywa	Tree	Adamson B6084 (EA)	Ripe fruits; edible.	[19,36,39,47]
Combretaceae					
^#^*Terminalia brownii* Fresen.	Muuku, kiuku	Shrub or tree	Gillett 19774 (EA)	Fruits; eaten by children.	[18,39,47]
Commelinaceae					
^#^*Commelina africana* L.	Kikowe	Herb	[36]	Leaves; eaten as a vegetable.	[20,28,36]
*Commelina benghalensis* L.	Itula	Herb	[36]	Leaves; eaten as a vegetable.	[36]
^#^*Commelina forskaolii* Vahl	Kikowe, kikoe	Herb	[36]	Leaves; eaten as a vegetable.	[15,36]
Compositae					
* *Launaea cornuta* (Hochst. ex Oliv. and Hiern) C.Jeffrey	Uthunga, muthunga	Herb	SAJIT-Mutie MU0209 (EA)	Leaves; eaten as a vegetable.	[22,36]
*Cyanthillium cinereum* (L.) H.Rob.	_	Herb	Kuchar 15163 (EA)	Leaves; eaten as a vegetable.	[36]
*Galinsoga parviflora* Cav.	_	Herb	Sheldrick TNP/E/109 (EA)	Leaves; eaten as a vegetable.	[36]
Convolvulaceae					
*Ipomoea lapathifolia* Hallier f.	Nzola, kinzola	Herb	Ossent 441A (EA)	Roots; tubers eaten raw.	[36]
*Ipomoea mombassana* Vatke	Ukwai wa nthi, wimbia, musele, uthui	Climber	Napper 1591 (EA)	Leaves; eaten as a vegetable.	[36]
Cucurbitaceae					
^#^*Momordica spinosa* Chiov.	_	Liana	Kuchar 14829 (EA)	Ripe fruits; edible.	[39,47]
* *Coccinia grandis* (L.) Voigt	Kimuya, kimowe, imore, imondiu	Climber	SAJIT-Mutie MU0176 (HIB)	Leaves, fruits; leaves eaten as a vegetable. Ripe fruits eaten raw or ground into flour and used to make porridge.	[36]
^#^* *Cucumis dipsaceus* Ehrenb. ex Spach	Kikungi, kyambatwa	Climber	SAJIT-Mutie MU0145 (HIB)	Leaves; eaten as a vegetable.	[36]
* *Kedrostis pseudogijef* C. Jeffrey	Mukauw’u	Climber	SAJIT-Mutie MU0212 (EA)	Leaves, fruits; leaves eaten as a vegetable. Ripe fruits are edible.	[36]
*Kedrostis gijef* C. Jeffrey	Witulu	Climber	Kuchar 1503 (EA)	Leaves, fruits; leaves eaten as a vegetable. Ripe fruits are edible.	[36]
* *Momordica rostrata* A. Zimm.	Kiongoa, kyongoa	Climber	SAJIT-Mutie MU0149 (HIB)	Leaves, seeds, fruits; ripe fruits are edible. Roasted seeds are edible. Leaves eaten as a vegetable.	[36]
^#^*Lagenaria siceraria* (Molina) Standl.	Ungu, kikuu, yungu	Climber	[36]	Leaves, fruits, seeds; young fruits edible when cooked. Seeds roasted and eaten. Leaves eaten as a vegetable.	[28,36]
^#^*Citrullus lanatus* (Thunb.) Matsum. and Nakai	Itikitiki	Climber	[28]	Fruits, leaves, seeds; ripe fruits are edible. Dry seeds ground into flour, mixed with sorghum flour and used to make porridge. Leaves eaten as a vegetable.	[28,36]
*Peponium vogelii*	_	Climber	Kimani 80 (EA)	Ripe fruits; edible.	[19]
Cyperaceae					
*Cyperus blysmoides* Hochst. ex C.B.Clarke	_	Herb	Edwards 23 (EA)	Roots; bulbs and stem bases eaten raw.	[20,28]
*Cyperus rotundus* L.	_	Herb	Porter 51 (EA)	Roots; stem bases are edible.	[36]
*Kyllinga alba*	_	Herb	Kuchar 8848 (EA)	Root bulbs; edible.	[19]
Ebenaceae					
^#^* *Diospyros mespiliformis* Hochst. ex A.DC.	Mukongoo ‘African ebony’	Tree	SAJIT-Mutie MU0179 (HIB)	Ripe fruits; edible.	[18,36,39,47]
*Euclea divinorum* Hiern	Mukinyai, mukinyai, mukuthi, nginyai	Shrub or tree	Kuchar 15097 (EA)	Fruits, bark; ripe fruits are edible. Bark added to soup as an appetizer.	[18,36,39]
Euphorbiaceae					
^#^* *Croton dichogamus* Pax	Mwalula, muthiani	Shrub or tree	SAJIT-Mutie MU0245 (EA)	Bark; used as a flavor in soup.	[18,20,39]
*Euphorbia schefleri* Pax	_	Shrub or tree	SAJIT-Mutie MU0188 (HIB)	Stems; smoke from wood used as a meat tenderizer.	[39]
Geraniaceae					
*Pelargonium quinquelobatum* Hochst. ex Rich.	_	Herb	Muasya 2459 (EA)	Stems; eaten raw.	[19]
Lamiaceae					
^#^* *Vitex payos* (Lour.) Merr.	Kimuu, muu	Shrub or tree	SAJIT-Mutie MU0286 (EA, HIB)	Ripe fruits; edible.	[36,39,47]
* *Vitex strickeri* Vatke and Hildebr.	Mwalika	Shrub or liana	SAJIT-Mutie MU0264 (EA)	Ripe fruits; edible.	[39]
* *Hoslundia opposita* Vahl	Musovi, musovasovi	Shrub	SAJIT-Mutie MU0244 (EA)	Fruits, leaves, stems; ripe fruits are edible. Leaves and stems used in tea.	[19,36,39]
*Ocimum basilicum* L.	Mutaa	Herb	[48]	Leaves; used for flavoring tea.	[18,36]
*Ocimum kilimandscharicum* Gürke	Wenye	Herb or shrub	Brilloe B303 (EA)	Leaves; used for flavoring tea.	[36,39]
*Ocimum gratissimum* L.	Mukandu	Shrub	Mbonge 6 (EA)	Leaves; used for flavoring tea.	[36,39]
* *Premna oligotricha* Baker	Mukaakaa	Shrub	SAJIT-Mutie MU0183 (EA)	Ripe fruits; edible.	[19]
^#^*Premna resinosa* (Hochst.) Schauer		Shrub	Kirika et al., NMK455 (EA)	Ripe fruits; edible.	[19,47]
^#^*Vitex doniana* Sweet	Kimuu ‘Black plum, vitex’	Tree	[18]	Ripe fruits; edible.	[18,36,39]
Leguminosae					
^#^* *Acacia nilotica* (L.) Delile	Musemei, musemeli	Tree	SAJIT-Mutie MU0224 (EA)	Bark, fruit; bark and pods boiled with sugar to make tea. Pods eaten during famine.	[18,36,39]
*Acacia reficiens* Wawra	_	Shrub or tree	Ament and Magogo 418 (EA)	Sweet inner bark; chewed raw.	[19]
^#^* *Acacia senegal* (L.) Willd.	King’olola	Shrub or tree	SAJIT-Mutie MU0122 (HIB)	Exudate; produces an edible gum.	[18,19,36,49]
^#^*Acacia seyal* Delile	Kinyua, kisewa	Shrub or tree	Robertson 4288 (EA)	Exudate, bark; produces an edible gum. Bark chewed raw or ground into powder to make tea.	[18,36,39,49]
*Acacia gerrardii* Benth.	Munina, kithi, muthii	Shrub or tree	[18]	Bark; used to make soup.	[18,39]
*Acacia drepanolobium* Sjostedt	Kiunga, muuga	Shrub or tree	[36]	Galls, fruits; inner flesh of the galls is edible. Young fruits are edible.	[36,39]
*Acacia hockii* De Wild.	Muuga, kinyua ‘white thorn’	Shrub or tree	Gardner 1088 (EA)	Exudate, bark; produces an edible gum. Inner bark chewed raw to quench thirst.	[36,39]
* *Acacia tortilis* (Forssk.) Hayne	Mwaa, kilaa, mulaa, muaa, ulaa	Tree	Sangai 935 (EA)	Exudate, fruits; produces an edible gum. Ripe pods eaten or ground into flour which is mixed with tea or blood.	[18,36,39]
*Albizia amara* (Roxb.) B.Boivin	Mwowa, muundua, kiundua, muundua	Tree	[36,48]	Exudate, stems; produces an edible gum. Dried stems used as an additive in food or soup and as a meat tenderizer.	[36,39]
*Bauhinia thonningii* Schum.	Mukolokolo	Shrub or tree	[36,48]	Fruits, leaves; dry fruit pulp is edible. Young sour shoots used in porridge or chewed raw.	[18,36]
*Crotalaria brevidens* var. *parviflora* (Baker f.) Polhill	Kamusuusuu	Herb	[50]	Leaves; eaten as a vegetable.	[36]
*Eriosema shirense* Baker f.	Ng’athu	Herb	[36]	Roots tubers; edible.	[36]
*Craibia laurentii* De Wild.	_	Tree	Mwachala et al., 487 (EA)	Seeds; beans eaten after boiling for several hours.	[19]
^#^*Vigna membranacea* A.Rich.	Ithookwe	Climber	Gillett 19475 (EA)	Roots, leaves; leaves eaten as a vegetable. Roots eaten raw or roasted.	[19,20,36]
*Vigna frutescens* A.Rich.	_	Climber	Bally B1536 (EA)	Root tubers; eaten raw or roasted.	[19,20,36]
*Vigna praecox* Verdc.	_	Climber	SAJIT-Mutie MU0115 (HIB)	Roots; boiled or roasted and eaten.	[19]
^#^* *Tamarindus indica* L.	Kithumula, muthumula, kikwasu, nthumula, nzumula, ngwasu	Tree	SAJIT-Mutie MU0208 (EA)	Fruits, leaves, seeds; fruit pulp eaten raw or used as a flavor in porridge or beer. Young leaves chewed raw or cooked as a vegetable. Seeds fried and eaten.	[18,22,36,39]
*Tylosema fassoglensis* (Schweinf.) Torre and Hillc.	Ivole	Climber	Hucks and Hucks 217 (EA)	Seeds, pods; seeds eaten raw, roasted or used as a coffee substitute. Unripe pods eaten raw.	[36]
^#^*Vatovaea pseudolablab* (Harms) J.B.Gillett	Kilukyo	Shrub or liana	[36]	Roots, leaves, flowers, pods, seeds; tubers cooked or roasted for food or eaten raw to quench thirst. Seeds eaten raw or cooked. Roots ground into flour and used for making porridge. Immature leaves, flowers and pods cooked as vegetables.	[19,20,36,39]
^#^*Cajanus cajan* (L.) Millsp.	Nzuu	Shrub	[36]	Seeds; cooked and eaten.	[22,36,51]
*Lablab purpureus* (L.) Sweet	Mbumbu, ngiima, nzavi	Climber	[36]	Seeds, leaves; beans cooked and eaten. Leaves eaten as a vegetable.	[19,36]
* *Vigna vexillata* (L.) A.Rich.	_	Climber	SAJIT-Mutie MU0257 (EA, HIB)	Roots; chewed raw to quench thirst.	[20]
^#^*Vigna unguiculata* (L.) Walp.	Nzooko, nthooko	Climber	[36]	Leaves; eaten as a vegetable.	[22,28]
*Ormocarpum kirkii* S.Moore	Muthingii	Shrub or tree	[48]	Leaves; eaten as a vegetable.	[18,22,39]
*Albizia anthelmintica* Brongn.	Mwowa, kyalundathi, kyowa kisamba	Shrub or tree	SAJIT-Mutie MU0194 (EA)	Leaves; eaten as a vegetable.	[22,39]
Loganiaceae					
^#^* *Strychnos decussata* (Pappe) Gilg	Mutolongwe	Shrub or tree	SAJIT-Mutie MU0109 (EA)	Ripe fruits; edible.	[39,47]
* *Strychnos henningsii* Gilg	Muteta	Shrub or tree	SAJIT-Mutie MU0200 (EA)	Roots, stems, bark, fruits; roots, stems and bark added to soup as a flavor. Fruits used for flavoring beer.	[18,36,39]
* *Strychnos spinosa* Lam.	Kyae, kimee, mae	Shrub or tree	SAJIT-Mutie MU0162 (EA)	Ripe fruits; edible.	[36,39]
Malvaceae					
^#^* *Azanza garckeana* (F.Hoffm.) Exell and Hillc.	Kitotoo, Mutoo	Tree	SAJIT-Mutie MU0289 (EA, HIB)	Ripe fruits; edible.	[18,36,39]
^#^* *Grewia tephrodermis* K.Schum.	Mulawa, kikalwa, ngalwa, ilawa	Shrub or tree	SAJIT-Mutie MU0220 (EA)	Ripe fruits; edible.	[18,19,36,39,47]
^#^* *Grewia villosa* Willd.	Muvu	Shrub	SAJIT-Mutie MU0206 (EA)	Ripe fruits; edible.	[18,19,36,39,47]
^#^*Grewia mollis* Juss.	_	Shrub or tree	Thomas 671 (EA)	Ripe fruits; edible.	[39,47]
* *Grewia arborea* (Forssk.) Lam.	Nguni	Shrub or tree	SAJIT-Mutie MU0321 (EA)	Ripe fruits; edible.	[39]
* *Grewia forbesii* Harv. ex Mast.	Mutalenda	Shrub, liana, tree	SAJIT-Mutie MU0270 (HIB)	Ripe fruits; edible.	[36,39]
*Grewia lilacina* K.Schum.	_	Shrub	Kirika et al., NMK462 (EA)	Fruits; edible.	[19]
*Grewia similis* K.Schum.	Mutuva	Shrub or liana	Edwards 681 (EA)	Ripe fruits; edible.	[19,36,39]
* *Grewia tembensis* Fresen.	Mutuva, nduva	Shrub	SAJIT-Mutie MU0242 (EA)	Ripe fruits; edible.	[19,36,39]
^#^*Grewia tenax* (Forssk.) Fiori	_	Shrub	Kirika et al., NMK457 (EA)	Ripe fruits; edible.	[19,36,39,47]
*Grewia trichocarpa* Hochst. ex A.Rich.	_	Shrub or tree	Lind and Agnew 5656 (EA)	Ripe fruits; edible.	[19,39]
*Hibiscus greenwayi* Baker f.	_	Shrub	[52]	Leaves, stems; young leaves eaten raw. Sweet stems chewed raw.	[19]
^#^*Adansonia digitata* L.	Kiamba, muamba	Tree	Bally 11691 (EA)	Roots, leaves, seeds; Root tips eaten during famine. Roots of germinating seeds are edible. Young leaves eaten as a vegetable. Roasted seeds are edible. Seed pulp eaten raw or boiled and the juice used as a sauce or added to porridge.	[18,20,36,39,47]
^#^*Corchorus olitorius* L.	_	Herb	[15]	Leaves; eaten as a vegetable.	[15,22]
* *Corchorus trilocularis L.*	_	Herb	SAJIT-Mutie MU0134 (EA)	Leaves; eaten as a vegetable.	[36]
* *Corchorus tridens* L.	_	Herb	SAJIT-Mutie MU0133 (HIB)	Leaves; eaten as a vegetable.	[22]
*Sterculia stenocarpa* H.J.P.Winkl.	_	Shrub or tree	Joana 7411 (EA)	Fruits; edible.	[19]
Moraceae					
* *Dorstenia hildebrandtii* var. *schlechteri* (Engl.) Hijman	_	Herb	SAJIT-Mutie MU0281 (EA, HIB)	Roots; eaten raw.	[19]
*Ficus capreifolia* Delile	_	Shrub or tree	Adamson 19716 (EA)	Ripe fruits; edible.	[39]
* *Ficus glumosa* Delile	Kionywe	Shrub or tree	SAJIT-Mutie MU0259 (EA, HIB)	Ripe fruits; edible.	[19,39]
*Ficus populifolia* Vahl	_	Shrub or tree	Gillett 18574 (EA)	Ripe fruits; edible.	[39]
^#^* *Ficus sycomorus* L.	Mukuyu	Tree	SAJIT-Mutie MU0202 (EA)	Ripe fruits; figs eaten or dried and made into flour which is mixed with maize flour for making porridge.	[18,19,36,47]
*Ficus sur* Forssk	_	Tree	[48]	Ripe fruits; edible.	[19,39,48]
*Ficus vasta* Forssk.	Mumbu, mukuyu	Tree	[48]	Ripe fruits; edible.	[39,48]
Menispermaceae					
*Chasmanthera dependens* Hochst.	Uswe	Liana	SAJIT-Mutie MU0039 (EA)	Roots, stems; roots boiled in milk as a drink for a child. Stems are edible.	[19,20,39]
Nymphaeaceae					
* *Nymphaea nouchali* var*. caerulea* (Savigny) Verdc.	_	Herb	SAJIT-Mutie MU0186 (EA)	Roots, flowers, fruits, seeds; edible.	[20,36]
Olacaceae					
^#^*Ximenia americana* L.	Kitula, mutula	Shrub or tree	[36]	Fruits, bark; ripe fruits are edible. Root bark used for tea.	[18,19,36,39,47]
*Jasminum abyssinicum* Hochst. ex DC.	Mukaksu	Climber	SAJIT-Mutie MU0154 (HIB)	Roots; roots boiled in broth or soup.	[20,39]
*Olea europaea* L.	Muthata, molialundi	Shrub or tree	[18]	Ripe fruits; edible.	[18,39]
*Olea capensis* L.	‘Elgon Olive, East African Olive’	Tree	[18]	Ripe fruits; edible.	[39]
Opiliaceae					
^#^*Opilia campestris* Engl.	Kiburuburu, mubrubru	Shrub	[18]	Ripe fruits; edible.	[18,19,39,47]
Pedaliaceae					
* *Sesamum calycinum* Welw.	Luta	Herb	SAJIT-Mutie MU0081 (EA)	Leaves; eaten as a vegetable.	[36]
Phyllanthaceae					
*Antidesma venosum* E.Mey. ex Tul.	Mukala, kitelanthia, kitolanthia	Shrub or tree	[36]	Ripe fruits; edible.	[36,39]
*Bridelia scleroneura* Müll.Arg.	_	Shrub or tree	Bally 1567 [42]	Ripe fruits; edible.	[39]
^#^* *Bridelia taitensis* Vatke and Pax ex Pax	Yathia, muandi, mwaanzia	Shrub or tree	SAJIT-Mutie MU0039 (EA)	Ripe fruits; edible.	[18,36,39,47]
^#^*Flueggea virosa* (Roxb. ex Willd.) Royle	Mukuluu, mukururu	Shrub	[48]	Ripe fruits; edible.	[18,19,36,39]
Poaceae					
*Dactyloctenium aegyptium* (L.) Willd.	Ukuku	Herb	[36]	Roots, seeds; rhizomes chewed raw. Grains ground into flour or chewed raw.	[36]
*Dactyloctenium giganteum* B.S.Fisher and Schweick.	Ukuku	Herb	[36]	Seeds; grains ground into flour for making porridge.	[36]
Polygonaceae					
*Oxygonum sinuatum* (Hochst. and Steud ex Meisn.) Dammer	Song’e	Herb	Bally 13179 (EA)	Leaves; eaten as a vegetable or chewed raw.	[19,36]
Portulacaceae					
*Portulaca oleracea* L.	Kamama, kamumama, kinyukwi	Herb	[36]	Leaves, seeds; leaves and slender stems eaten raw or cooked as a vegetable. Seeds ground into flour for making porridge.	[36]
* *Portulaca quadrifida* L.	Kenyinyia, kamumama	Herb	SAJIT-Mutie MU0121 (EA)	Leaves, seeds; leaves and slender stems eaten raw or cooked as a vegetable. Seeds ground into flour for making porridge.	[36]
Putranjivaceae					
*Drypetes gerrardii* Hutch.	_	Tree	Burry 4 (EA)	Fruits; eaten raw.	[19]
Rhamnaceae					
* *Berchemia discolor* (Klotzsch) Hemsl.	Kisanawa, kisaaya, nzaaya, nzanawa	Shrub or tree	SAJIT-Mutie MU0293 (EA)	Fruits, exudate; ripe fruits are edible. Produces an edible gum.	[18,19,36,39]
^#^*Ziziphus mucronata* Willd.	Kitola usuu, kitolousuu, muae	Shrub or tree	[36,48]	Bark, fruits; ripe fruits are edible. Bark used in tea.	[18,19,36,39]
^#^*Scutia myrtina* (Burm.f.) Kurz	Mtanda mboo, kitumbuu, mbombo	Shrub or tree	[36]	Roots, fruits; ripe fruits are edible. Roots used in soup.	[18,19,36,39,47]
*Ziziphus abyssinica* Hochst. ex A.Rich.	Muae, kitolousuu	Shrub, liana or tree	[53]	Ripe fruits; edible.	[19,39]
*Ziziphus pubescens* Oliv.	_	Shrub or tree	[42]	Ripe fruits; edible.	[36,39]
*Ziziphus jujuba* Mill.	_	Shrub or tree	[36]	Ripe fruits; edible and made into flour.	[22,36,39]
Rubiaceae					
*Canthium glaucum* Hiern	_	Shrub or tree	[36]	Ripe fruits; edible.	[36]
*Pavetta gardeniifolia* Hochst. ex A.Rich.	_	Shrub	[54]	Fruits; edible.	[19]
^#^* *Rothmannia urcelliformis* (Hiern) Bullock ex Robyns	Mutendeluka	Shrub or tree	SAJIT-Mutie MU0164 (HIB)	Ripe fruits; edible.	[18]
^#^* *Vangueria madagascariensis* J.F.Gmel.	Kikomoa, mukomoa	Shrub or tree	SAJIT-Mutie MU0280 (EA, HIB)	Ripe fruits; edible and used for flavoring beer.	[18,19,36]
*Rothmannia fischeri* (K.Schum.) Bullock ex Oberm.	Muendeluka	Shrub or tree	Owino and Mathenge 214 (EA)	Ripe fruits; edible.	[39]
^#^* *Tennantia sennii* (Chiov.) Verdc. and Bridson	Kisilingu	Shrub	SAJIT-Mutie MU0207 (EA)	Ripe fruits; edible.	[39,47]
^#^*Vangueria infausta* Burch.	Kikomoa, mukomoa, muteleli	Shrub or tree	Joana B1142 (EA)	Ripe fruits; edible.	[36,39,47]
*Vangueria volkensii* K.Schum.	Kikomoa, mukomoa	Shrub or tree	Gibbons OX635 (EA)	Ripe fruits; edible.	[36,39]
^#^* *Vangueria schumanniana* (Robyns) Lantz	Mukomole, kitootoo, ngomole, ndootoo	Shrub	Napper 1590 (EA)	Fruits, stems; ripe fruits are edible. Stems smoked and inserted into gourds of milk to induce good flavor in milk.	[36,39,47]
*Vangueria apiculata* K.Schum.	Kikomoa, mukomoa	Shrub or tree	[52]	Fruits; edible and used for flavoring beer.	[36,39]
* *Meyna tetraphylla* (Schweinf. ex Hiern) Robyns	Kitotoo, kitootoo, kakomoa, kitolousuu	Shrub or tree	Bally 1636 [42]	Ripe fruits; edible.	[36,39]
Rutaceae					
^#^* *Zanthoxylum chalybeum* Engl.	Mukenea, mukanu	Shrub or tree	SAJIT-Mutie MU0317 (EA)	Bark, leaves, fruit; bark and fruits used as food spices. Leaves and fruits used in flavoring tea. Bark used in making or flavoring tea.	[18,36,39]
*Vepris glomerata* Engl.	_	Shrub or tree	Trapnell 2406; Thomas 673 [42]	Ripe fruits; edible.	[39]
* *Vepris simplicifolia* (Engl.) Mziray	Mutuyu	Shrub or tree	SAJIT-Mutie MU0234 (EA, HIB)	Fruits; edible.	[19]
*Harrisonia abyssinica* Oliv.	Mkiliulu	Shrub or tree	Mutie MU0185 (HIB)	Ripe fruits; edible.	[18,39]
* *Zanthoxylum holtzianum* (Engl.) P.G. Waterman	_	Shrub or tree	SAJIT-Mutie MU0180 (HIB)	Ripe fruits; edible.	[22]
Salicaceae					
*Flacourtia indica* (Burm.f.) Merr.	Kiathani, kikathani	Shrub or tree	Festo and Luke 2291 (EA)	Ripe fruits; edible.	[36,39]
Salvadoraceae					
^#^*Dobera glabra* (Forssk.) Juss. ex Poir.	Kisiu, kithio, kikaitha	Shrub or tree	Edwards EAH 12315 [42]	Exudate, fruits, seeds; produces an edible gum. Ripe fruits are edible. Boiled seeds are edible.	[18,36,39,47]
^#^*Salvadora persica* L.	Mukayau	Shrub or tree	Pearce 405 (EA)	Ripe fruits; edible.	[18,19,36,39,47]
Santalaceae					
*Osyris lanceolata* Hochst. and Steud.	Kithawa	Shrub or tree	Birch 59/13 (EA)	Ripe fruits; edible.	[39]
Sapindaceae					
*Allophylus africanus* P.Beauv.	_	Shrub or tree	SAJIT-Mutie MU0165 (HIB)	Fruits; edible.	[19]
^#^*Pappea capensis* Eckl. and Zeyh.	Kyuua, kiva, mba	Shrub or tree	[18,36]	Fruits, bark; ripe and unripe fruits are edible. Dry inner bark used for tea.	[18,36,39,47]
*Haplocoelum foliolosum* (Hiern) Bullock	Mukumu, mukumi	Shrub or tree	[54]	Ripe fruits; edible.	[18,39]
Sapotaceae					
^#^*Manilkara mochisia* (Baker) Dubard	Kinako, kisaa	Tree	[36]	Ripe fruits; edible.	[18,36,47]
Solanaceae					
^#^*Solanum americanum* Mill.	Kitulu	Herb	[36]	Leaves; eaten as a vegetable.	[21,22,36]
Talinaceae					
* *Talinum portulacifolium* (Forssk.) Asch. ex Schweinf.	_	Herb	SAJIT-Mutie MU0014 (EA)	Leaves; eaten raw.	[19]
Verbenaceae					
*Lantana camara* L.	Kitavisi, mukiti, musomolo	Shrub	[36]	Ripe fruits; edible.	[36,39]
*Lantana humuliformis* Verdc.	_	Shrub	Kuchar 14908 (EA)	Ripe fruits; edible.	[36]
*Lantana ukambensis* (Vatke) Verdc.	_	Herb	Napier 1567 (EA)	Ripe fruits; edible.	[36]
*Lippia javanica* (Burm.f.) Spreng.	Muthiethi	Shrub	[18]	Fruits, leaves; ripe fruits are edible. Leaves used for tea.	[18,36]
*Lippia kituiensis* Vatke	Muthiethi, muthiiti, muthyeti	Shrub	[36]	Fruits, leaves; ripe fruits edible. Leaves used for tea.	[36,39]
Vitaceae					
*Cissus aphyllantha* Gilg	Mwelengwa	Shrub or liana	SAJIT-Mutie MU0247 (EA)	Ripe fruits; edible.	[39]
*Cissus rotundifolia* Vahl	Itulu	Shrub	SAJIT-Mutie MU0128 (EA)	Ripe fruits; edible.	[39]
^#^*Cyphostemma adenocaule* (Steud. ex A.Rich.) Desc. ex Wild and R.B.Drumm.	_	Climber	SAJIT-Mutie MU0143 (EA)	Leaves; eaten as a vegetable.	[22]
Xanthorrhoeaceae					
* *Aloe secundiflora* Engl.	Kiluma	Herb	SAJIT-Mutie MU0191 (EA)	Roots, flowers, peduncle; roots used to ferment traditional beer. Flower nectar is edible. Sweet base of inflorescence is chewed raw.	[36]
Zygophyllaceae					
^#^* *Balanites aegyptiaca* (L.) Delile	Kilului, kiluluwi, mulului	Tree	SAJIT-Mutie MU0196 (EA)	Exudate, fruits, leaves, seeds; produces an edible gum. Ripe fruits are edible. Leaves and tender shoots eaten as a vegetable. Inner part of a seed is edible when boiled.	[18,36,39,47]
*Balanites glabra* Mildbr. and Schltr.	Kilului	Shrub or tree	[48]	Ripe fruits; edible.	[39]
*Balanites pedicellaris* Mildbr. and Schltr.	_	Shrub or tree	[36]	Seeds, fruits; ripe fruits are edible. Inner part of the seed cooked and eaten.	[36,39]
*Balanites rotundifolia* (Tiegh.) Blatt.	Kilului	Shrub or tree	[36]	Fruit, seeds; fruit pulp is edible and used to make local brew. Inner part of seed is edible when cooked.	[36,39]
*Balanites wilsoniama* Dawe and Sprague	Kivuw’a	Tree	[32]	Ripe fruits; edible.	[36]
